# Fiber Bragg Grating Dilatometry in Extreme Magnetic Field and Cryogenic Conditions

**DOI:** 10.3390/s17112572

**Published:** 2017-11-08

**Authors:** Marcelo Jaime, Carolina Corvalán Moya, Franziska Weickert, Vivien Zapf, Fedor F. Balakirev, Mark Wartenbe, Priscila F. S. Rosa, Jonathan B. Betts, George Rodriguez, Scott A. Crooker, Ramzy Daou

**Affiliations:** 1 National High Magnetic Field Laboratory, Los Alamos National Laboratory, Los Alamos, NM 87545, USA; vzapf@lanl.gov (V.Z.); fedor@lanl.gov (F.F.B.); jbbetts@lanl.gov (J.B.B.); crooker@lanl.gov (S.A.C.); 2 Institute for Materials Science, Los Alamos National Laboratory, Los Alamos, NM 87545, USA; carolinacorvalan@gmail.com; 3 Gerencia de Materiales, Comisión Nacional de Energia Atómica, Avda. Gral. Paz 1499, B1650KNA San Martín, Buenos Aires, Argentina; 4 Consejo Nacional de Investigaciones Científicas y Técnicas, Godoy Cruz 2290, C1425FQB Ciudad Autónoma de Buenos Aires, Argentina; 5 Universidad Nacional Tres de Febrero, Valentín Gómez 4828, Caseros, B1678ABJ Buenos Aires, Argentina; 6 National High Magnetic Field Laboratory, Florida State University, Tallahassee, FL 32310, USA; weickert@lanl.gov (F.W.); mrw03h@fsu.edu (M.W.); 7 Condensed Matter and Magnet Science Group, Materials, Physics, and Applications Division, Los Alamos National Laboratory, Los Alamos, NM 87545, USA; pfsrosa@lanl.gov; 8 Center for Integrated Nanotechnologies Group, Materials, Physics, and Applications Division, Los Alamos National Laboratory, Los Alamos, NM 87545, USA; rodrigeo@lanl.gov; 9 Laboratoire de Cristallographie et Sciences des Matériaux, Normandie Université, Ecole Nationale Supérieure d'Ingénieurs de Caen, Université de Caen Normandie, Centre National de la Recherche Scientifique, 14050 Caen, France; ramzy.daou@ensicaen.fr

**Keywords:** single-mode fiber Bragg gratings, FBG, large pulsed magnetic fields, superconducting magnets, magnetostriction, thermal expansion, quantum oscillations, phase transitions

## Abstract

In this work, we review single mode SiO_2_ fiber Bragg grating techniques for dilatometry studies of small single-crystalline samples in the extreme environments of very high, continuous, and pulsed magnetic fields of up to 150 T and at cryogenic temperatures down to <1 K. Distinct millimeter-long materials are measured as part of the technique development, including metallic, insulating, and radioactive compounds. Experimental strategies are discussed for the observation and analysis of the related thermal expansion and magnetostriction of materials, which can achieve a strain sensitivity (*ΔL/L*) as low as a few parts in one hundred million (≈10^−8^). The impact of experimental artifacts, such as those originating in the temperature dependence of the fiber’s index of diffraction, light polarization rotation in magnetic fields, and reduced strain transfer from millimeter-long specimens, is analyzed quantitatively using analytic models available in the literature. We compare the experimental results with model predictions in the small-sample limit, and discuss the uncovered discrepancies.

## 1. Introduction

Bragg gratings are inscribed over a length of an optical fiber, normally tuned to reflect a particular wavelength of infrared light used in telecommunication. As strain sensors, the Bragg-reflected wavelength monitors the spacing of the grating, and hence provides a measure of strain along the length of the sensitive region with *ΔL/L* ~ 10^−8^ precision. As a result, fiber Bragg gratings (FBGs) are well suited for sensing applications and have been used to measure dilation, temperature, pressure, gas (moisture) absorption/diffusion, among other properties [1,2,3,4,5]. FBGs are especially appropriate for sensing under extreme conditions due to their immunity to electromagnetic interference and the possibility for rapid and accurate interrogation. By attaching small FBGs to millimeter-size single-crystalline samples, one is able to detect small changes in sample length that are induced by extremely large magnetic fields (magnetostriction), covering the range from a few Tesla in superconducting magnets to 150 T in destructive pulsed electromagnets, or by changes in temperature (thermal expansion) at extremely low temperatures from room temperature down to *T* < 1 K. 

Thermal expansion and magnetostriction are fundamental thermodynamic quantities that are directly derived from the Gibbs free energy *G*(*p,T,H*) of materials. Changes in *G* caused by varying external parameters such as pressure (*p*), temperature (*T*), or magnetic field (*H*) can be studied by the thermal expansion coefficient *α* = *∂^2^G/∂p∂T* or the magnetostriction coefficient *λ* = *∂^2^G/∂p∂H*, respectively. Therefore, dilatometry techniques belong to the basic set of experimental probes present in materials science laboratories. These techniques are used alongside other fundamental magnetic, electric, and thermal capabilities to identify states of matter, to detect classical and quantum phase transitions between different ground states, and to understand the characteristics and nature of such transitions and transformations.

**Figure 1:**
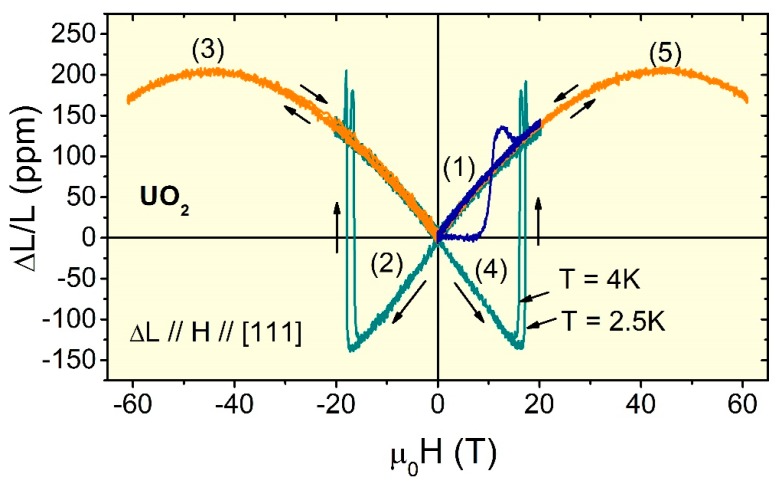
Magnetostriction vs. magnetic field for a single crystalline sample of UO_2_, measured at 2.5 K and 4 K during a 25 millisecond pulsed field exceeding 60 T. The magnetic field was applied along the [111] crystallographic direction in the sequence indicated by numbers (1) through (5). An irreversible transition is observed when the magnetic field is first applied to 20 T (1). Upon reversal of the magnetic field, the magnetostriction changes sign too (piezomagnetism) and the irreversible transition now is much sharper (2). Two consecutive pulses in the same direction superimpose with no irreversibilities (3). The magnetostriction *ΔL/L* changes sign again when the magnetic field direction is reversed to positive (4) and becomes reversible when a second pulse is applied in the same direction (5). These results first discovered piezomagnetism in UO_2_, a technologically important material and also the strongest piezomagnet known [23]. These data were obtained using the 46 kHz line array InGaAs camera described in Section 2.2 below. The *ΔL/L* resolution achieved in this experiment is approximately 2 × 10^−6^.

When compared to electrical transport or magnetic properties measurements for instance, dilatometry techniques have been slow in catching up with advances in the area of fast and ultrafast measurements, which are necessary when the timescales of extreme magnetic field pulses become as short as microseconds. Sensing of magnetostriction with single mode SiO_2_ FBGs in pulsed magnetic fields is successfully utilized at the National High Magnetic Field Laboratory (NHMFL) with a resolution as good as a few parts per hundred million (*ΔL/L* ≈ 10^−8^) in the best cases. This capability allows for the study of a variety of insulating and metallic condensed matter systems including geometrically frustrated magnets, quantum magnets, multiferroics, and uranium- and cerium-based antiferromagnets [6,7,8,9,10,11,12,13,14,15,16,17,18,19,20,21,22,23,24,25,26,27,28,29,30]. Figure 1 shows an example of magnetoelastic effects in pulsed magnetic fields to 60 T at cryogenic temperatures on a sample of uranium dioxide (UO_2_), which is the most commonly used nuclear fuel. The intriguing behavior exhibited by this material, i.e., a field-induced strain butterfly loop characteristic of piezomagnets, is due to its peculiar 3***k***-type antiferromagnetic state below *T* = 30 K that breaks time-reversal symmetry. The record high switching fields at ±18 T mark antiferromagnetic domains that flip [23]. In this review paper, we discuss experimental approaches that are used in three different timescales (milliseconds, microseconds, and nanoseconds), as well as the technique applicability, sensitivity, and advantages over traditional methods. We also consider various artifacts including non-uniform strain transfer from gluing procedures and reduced sample dimensions, and optical polarization (Faraday) rotation due to the large magnetic field.

## 2. Materials and Methods

Magnetostriction is most accurately measured in static magnetic fields using a capacitance dilatometer, where the change in length of the sample moves one plate of a parallel plate capacitor [30,31,32,33] with a sensitivity as good as *ΔL/L* = 10^−10^. A plastic-bodied device was previously tested in pulsed magnetic fields [34], but eddy currents in the relatively small capacitor plates, as well as the strong mechanical vibration that is induced by the field pulse limited the strain sensitivity to *ΔL/L* ~ 10^−5^. Other strain-sensitive techniques, such as those using resistive foil strain gauges [35], piezoresistors [36], and piezoresistive cantilevers [37] have been tested in similar environments with different degrees of success. The sensitivity of resistive strain gauges is, however, severely compromised by pervasive electromagnetic noise. The performance of piezomagnetic devices is, in turn, hindered by their strong temperature dependent elastic/electric properties, leading to strongly temperature dependent sensitivity, and intrinsic drift (time dependence) in their electrical properties. 

An optical FBG strain sensor has considerable advantages in this challenging environment. It is largely insensitive to electromagnetic interference, in particular electromagnetic induction and subsequent electromotive forces caused by the rapidly changing magnetic field, which can produce peaks of hundreds of volts on electrical wiring. Furthermore, their sensitivity to mechanical vibration can be reduced to much less than can be seen in capacitance dilatometers. This is an advantage shared by other strain gauges that are affixed directly to the sample. Because of the small diameter of optical fibers, however, a much smaller surface area is required than that for a classic resistive foil gauge. We estimate the force applied by a 125 μm telecom-type fiber on samples under study to be in the 1–3 Newton range (~1/20 of the force that can be applied with bare fingertips). Consequently, the sample is under very low applied stress from the bonding process, and smaller than traditional sample dimensions can be explored (see Section 4.4).

FBG interrogation systems can be constructed with a frequency response that is appropriate to the time scale of the experimentally produced magnetic fields [38,39,40,41,42]. Continuous magnetic fields in the range of tens of Tesla are produced with superconducting (20 T), resistive (35 T), and hybrid (45 T) electromagnets [40]. Interrogation times in the millisecond range are, in these cases, sufficient. Capacitor bank driven pulsed magnets to 100 T typically last tenths of milliseconds and require acquisition rates in the microsecond domain [38,39,41]. Magnetic fields beyond these values, to 250 T, can only be produced at present times with destructive single-turn type magnets [42]. Such magnetic pulses only last for a few microseconds and FBGs attached to materials must be interrogated as fast as every few nanoseconds. 

### 2.1. Fast (Millisecond Time Scale) FBG Interrogation Using Swept-Wavelength Lasers

**Figure 2:**
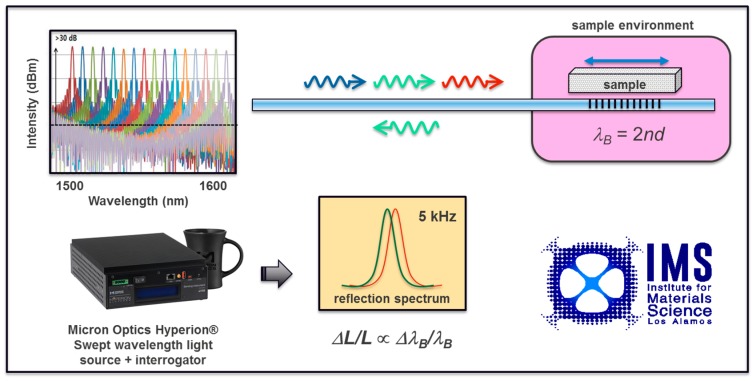
Experimental setup for fiber Bragg gratings (FBG) interrogation with a swept wavelength laser source, such as the Micron Optics Hyperion^®^ instrument. *λ_B_* is the Bragg wavelength, *n* is the fiber’s index of diffraction, and *d* is the grating spacing.

Swept wavelength laser systems based on tunable laser sources can be used in the 1500–1600 nm range to interrogate FBGs at frequencies up to 5 kHz, such as the commercially available Hyperion^®^ family of instruments that is manufactured by Micron Optics, Atlanta, GA, USA. The sampling rate permits convenient time averaging used to improve sensitivity, and a rapid response to phase transformations in the material or to a changing environment. This class of interrogators includes a light depolarization option that makes them suitable for high magnetic field environments, and is ideal for FBG based dilatometry in continuous magnetic fields and/or hydrostatic pressures [27]. We have implemented a compact and portable swept wavelength-based interrogation system. We successfully used it at the NHMFL for dilatometry studies of materials in superconducting (15 T), resistive (35 T) and hybrid (45 T) magnets at cryogenic temperatures down to 0.3 K (see Figure 2). The multiplexing capabilities of the detection chain allowed for the simultaneous monitoring of several samples and their respective reference channels. Moreover, the swept wavelength nature of the light source in this type of application implies extremely low power (0.06 mW), when compared to broadband optical sources in the same wavelength range, making this instrument an ideal companion in cryogenic temperatures. The sensitivity of FBG interrogation systems is directly impacted by the spectral width of the reflected light peak at the Bragg condition. As discussed below, the full width at half maximum (*FWHM*) of the peak reflected by the FBGs is inversely proportional to the FBG length, with a 2 mm long FBG (manufactured by Technica SA, Atlanta, GA, USA), typically reflecting a ≈ 1 nm wide peak. The peak detection algorithm implemented in the Hyperion^®^ family of instruments currently requires *FWHM* ≤ 1 nm, making measurements of samples smaller than 2 mm challenging. The sensitivity achieved with this method can reach a few parts in one hundred million (*ΔL/L* ≈ 10^−8^), as shown below, in Section 3.1. 

### 2.2. Very Fast (Microsecond Time Scale) FBG Interrogation Using Broadband Light and a Line Array Camera

**Figure 3:**
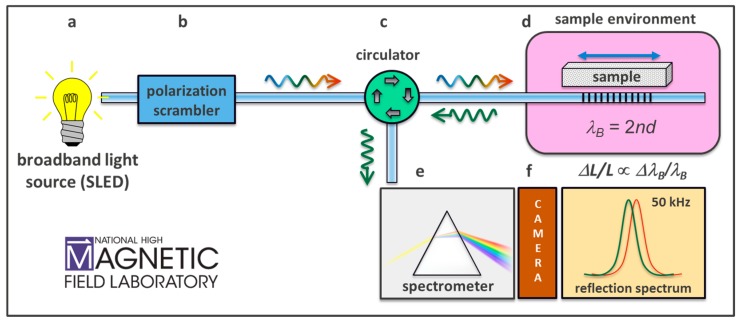
Experimental setup for high magnetic field FBG-based dilatometry implemented at the National High Magnetic Field Laboratory (**a**) Superluminescent broadband light emitting diode, operating in the 1500–1600 nm range. (**b**) Polarization scrambler. (**c**) Circulator. (**d**) Sample environment, in our case cryogenic temperatures to 0.5 Kelvin, and magnetic fields to 100 T. (**e**) Spectrometer and InGaAs line array combo, operating to 46 kHz. *λ_B_* is the Bragg wavelength, *n* is the fiber’s index of diffraction, and *d* is the grating spacing.

In our 46 kHz interrogation scheme, the FBG is illuminated by a broadband white light source in the infrared telecom spectrum (~1500–1600 nm) using a commercial superluminescent light-emitting diode (SLED). The narrow spectral band around 1550 nm that is reflected by the FBG is diverted via a circulator to a 0.5 m spectrometer, where it is spectrally dispersed and detected by an InGaAs linescan camera. A schematic of an improved version of the setup used by Daou et al. [6], including a light polarization scrambler, is shown in Figure 3. The theoretical limits on accuracy arise from the specifications of available components, such as the FBG length, spectrometer grating specs and camera dynamic range. For non-destructive pulsed fields (ms duration), line-array cameras that can capture the entire FBG reflection spectrum at rates of up to 150 kHz (time span between spectra <10 μs) are now available. The best signal-to-noise ratio is achieved by using the maximum necessary illumination so that the full dynamic range of the camera sensitivity is utilized. The strain sensitivity achieved with this approach can be as good as one part in 10 million (10^−7^), see examples below. In a more recent work, we addressed subtleties and constraints related to the optical readout and experimental environment, as described in Section 4.

### 2.3. Ultrafast (Nanosecond Time Scale) FBG Interrogation Using Pulsed Lasers

**Figure 4:**
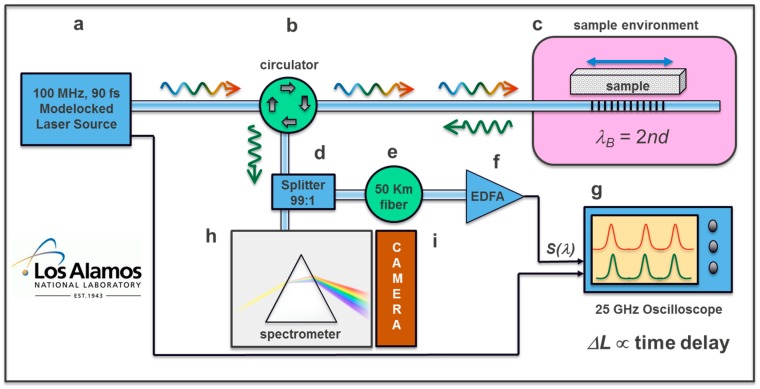
Coherent pulse FBG interrogation system working at 100 MHz [16,17]. (**a**) Pulsed laser light source, (**b**) circulator, (**c**) controlled sample environment in a 250 T single turn coil magnet, (**d**) 1 × 2 99:1 splitter sends 1% of the light to spectrometer, (**e**) 50–100 km spool of chromatic dispersive fiber, (**f**) Erbium-doped fiber amplifier, (**g**) ultrafast 25 GHz oscilloscope, (**h**) line array camera used for monitoring of the FBG reflection. λ_B_ is the Bragg wavelength, n is the fiber’s index of diffraction, and d is the grating spacing.

Magnetic fields in excess of 100 T can be achieved in the laboratory, but typically only for very short durations of a few microseconds, and typically resulting in the destruction of the magnet in the process. The so-called ‘single-turn magnets’ are examples of magnets of this type [42]. To measure magnetostriction using FBGs in such an environment, interrogation times of order 10 nanoseconds are desired, which is equivalent to an interrogation rate of 100 MHz. This greatly exceeds the readout rate of linescan cameras discussed in the previous section. Therefore, we turn to ultrafast optical techniques by using femtosecond pulsed lasers to map spectral shifts into the time domain. Figure 4 shows a schematic diagram of the approach. An ultrafast laser produces 90 fs optical pulses at a repetition rate of 100 MHz (10 ns between pulses). The pulses have a broad wavelength spectrum that is centered at 1560 nm, with a width of 100 nm (1510–1610 nm at the −10 dB points). A smaller part of this spectrum, ~1 nm wide, is reflected by the FBG. This reflected pulse is then directed through a very long (~50 km) optical fiber. Because of material dispersion in the optical fiber (i.e., the index of refraction of glass varies with wavelength), the arrival time of this reflected pulse depends sensitively on its center wavelength. In this way, spectral shifts are encoded onto temporal shifts of the pulse arrival time, which can be measured very accurately by using fast detectors and a fast (25 GHz) oscilloscope. Each pulse arrival constitutes an independent measurement, and therefore, an FBG interrogation rate of 100 MHz is achieved. A more detailed description of the ultrafast setup is published elsewhere [16,17]. This system has also been used to measure other fast processes such as explosive detonations and shock wave propagation [43]. The strain resolution achieved so far with this technique is better than one part in ten thousand (*ΔL/L* < 10^−4^). A variation of this method, with somewhat worse strain resolution, uses an optical filter to detect spectral shifts encoded onto pulse intensity [44]. 

## 3. A Review of Selected Recent Results

A number of materials of current scientific interest have recently been studied with FBG dilatometry in high magnetic fields at cryogenic temperatures. We discuss some representative examples in the following.

### 3.1. Magnetostriction Superstructure in the Frustrated Quantum Spin System SrCu_2_(BO_3_)_2_

**Figure 5:**
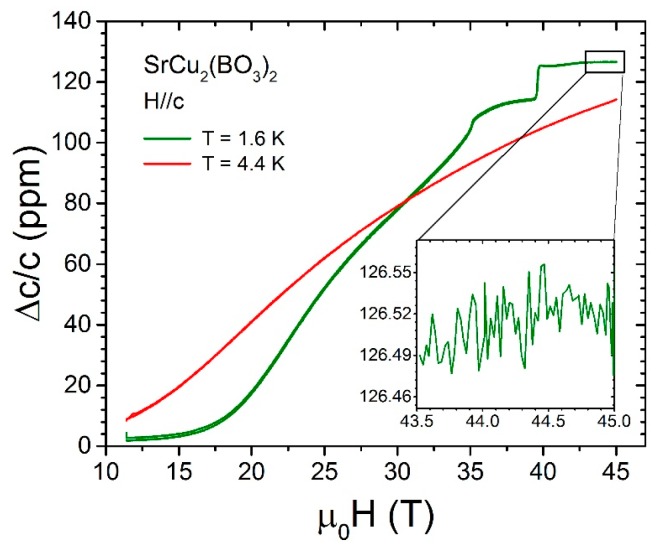
Axial magnetostriction along the crystallographic *c*-axis (Δ*c/c*) in the frustrated quantum magnet SrCu_2_(BO_3_)_2_ measured to 45 T at 1.6 K and 5.3 K taken with the Hyperion^®^ model si155 interrogator by Micron Optics in a continuous 45 T hybrid magnet at the National High Magnetic Field Laboratory (NHMFL). For these measurements, a 2 mm-long FBG manufactured by Technica SA was used. The magnetostriction plateaus observed at 35 T and 39.65 T correspond to magnetization plateaus observed at relative magnetization *M/Ms* = 1/4 and 1/3, respectively. The strain sensitivity achieved in this measurement is approximately 2 × 10^−8^ [30].

Quantum magnets, i.e., low-spin (*s* = 1/2 or *s* = 1) magnets with a gapped ground state, are excellent test beds for our current understanding of magnetism. These magnets may host magnetic moments in different types of geometry (dimers, triangular lattices, plaquettes, etc.), different dimensionalities (one dimensional, two dimensional, three dimensional), or different degrees of magnetic frustration that hinder magnetic ordering. In the case of SrCu_2_(BO_3_)_2_ the magnetic ions are Cu^2+^ (spin *s* = 1/2) arranged in orthogonal dimers placed on a square lattice. Layers of orthogonally coordinated dimers connected by BO_3_ complexes, and separated by Sr layers, form a quasi two-dimensional magnet. In spite of the Cu^2+^ magnetism, this material does not magnetically order at any temperature due to the formation of non-magnetic spin dimers between neighboring Cu^2+^ atoms and the concomitant spin energy gap that defines the ground state at cryogenic temperatures. External magnetic fields can be used, however, to induce a magnetic superstructure [11,15] that has a direct impact on the crystal lattice of the material. Figure 5 displays the magnetostriction of SrCu_2_(BO_3_)_2_ measured in continuous magnetic fields to 45 T in a hybrid continuous magnet at cryogenic temperatures. The technique used for these measurements is that described in Section 2.1, and the sensitivity achieved is *ΔL/L* ~ 2 × 10^−8^. 

### 3.2. Transverse Magnetostriction

**Figure 6:**
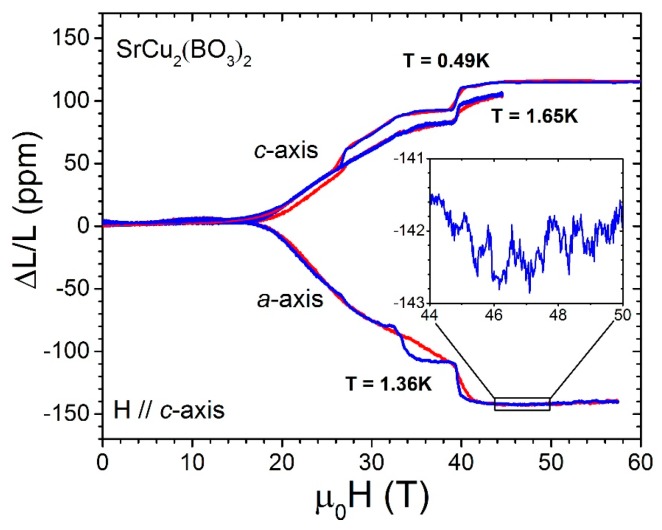
Transverse magnetostriction of SrCu_2_(BO_3_)_2_ measured at cryogenic temperatures below 2 K. The data were taken in a 60 T long-pulse magnet (2.5 s long) at the NHMFL using the method described in Section 2.2. Red curves were taken during magnetic field upsweep, blue curves during downsweep. These curves do not completely overlap due to cooling/heating caused by magnetocaloric effect. Superstructure in both axial and transverse magnetostriction is evident. The strain resolution achieved in these measurements is better than 10^−6^ [15] as shown in the inset.

The space available for experiments in a pulsed field cryostat has a typical diameter of a few mm. It is much more straightforward to have the FBG aligned longitudinally, in the same direction as the applied magnetic field, as the fiber can be kept straight. In order to be sensitive to strain that is transverse to the applied magnetic field, which is essential to the study of volume magnetostriction, the fiber must be bent at 90°. On one hand, the minimum bend radius is around 5 mm, and, from a mechanical stand point, such a sharp turn can cause leakage of >90% of the light from the fiber. On the other hand, bright light sources available (several mW) enable sensitive measurements even when a large fraction of the light escapes the fiber at the bent section. Successful measurements of volume magnetostriction have been reported for LaCoO_3_ [10], SrCu_2_(BO_3_)_2_ [15], β-TeVO_4_ [19], and URu_2_Si_2_ [29] single crystals. From our experience, it is possible to bend the fiber as long as the bent section is a few millimeters away from the sample position in such way that the section of fiber that contains the FBGs remains straight. Fiber bending close to the FBGs will, otherwise, cause background signal fluctuations [45,46] and severely reduced sensitivity. When the bend is implemented correctly, the sensitivity of the technique (*ΔL/L* < 10^−6^) is not compromised (see Figure 6).

### 3.3. Quantum Oscillations in the Magnetostriction of Metals

**Figure 7:**
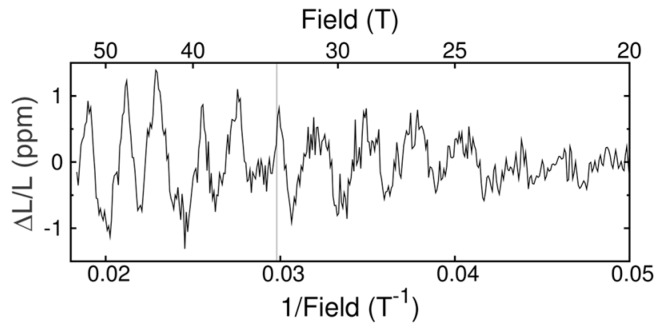
Magnetostrictive quantum oscillations in GdSb at 2 K taken in a 55 T mid-pulse magnet using the method described in Section 2.2. The resolution achieved in this measurement is considerably better than 10^−6^ [9].

Quantum oscillations arise when conductors are subjected to external magnetic fields such that the electronic states at the Fermi level become separated into Landau levels whose degeneracy and cyclotron orbit depend on the magnetic field. When the Landau level that is imposed by the magnetic field matches a closed orbit in the Fermi surface, a change occurs in nearly all of the properties of the material, including its length [47]. Scanning the magnetic field, thus, leads to oscillations in all thermodynamic and transport properties of a material. In some cases these oscillations are difficult to observe and can require a combination of clean samples, high magnetic fields, and low temperatures that are challenging to achieve. Access to very high fields is one way to resolve quantum oscillations that would otherwise be impossible to detect. The amplitude of quantum oscillations scales as the magnetic field square, making them easier to detect in high fields, while the mean free path of the sample can be shorter at higher fields, allowing the detection of quantum oscillations in dirtier samples. Magnetostriction in particular is a bulk property, and thus is less susceptibility to surface or impurity states than e.g., transport or magnetization. It is also easier to measure in high fields than heat capacity. Thus, it is a uniquely bulk probe of quantum oscillations. The need to distinguish between bulk, impurity, and surface states is of particular current relevance to the field of topological insulators. We observed quantum oscillations in the magnetostriction of a semimetal single crystal, GdSb, using the FBG technique in pulsed magnetic fields up to 55 T [9]. These data agree well with the quantum oscillations seen in the magnetization, although the phases and amplitudes of the oscillations are somewhat different. These differences are due the pressure dependence of individual Fermi surface sheets, and provide valuable complementary information about the electronic coupling and deformation potentials. Figure 7 shows a trace of the magnetostriction curve with a smooth background, which is obtained from a second order polynomial fit, subtracted. At 33 T the antiferromagnetic ground state is suppressed by the magnetic field and the quantum oscillations change character. The oscillations are easily distinguished from Faraday rotation artifacts discussed in Section 4.3 because they are periodic in inverse magnetic field, 1/B.

### 3.4. Spin Transitions in the 100 T Range in LaCoO_3_

**Figure 8:**
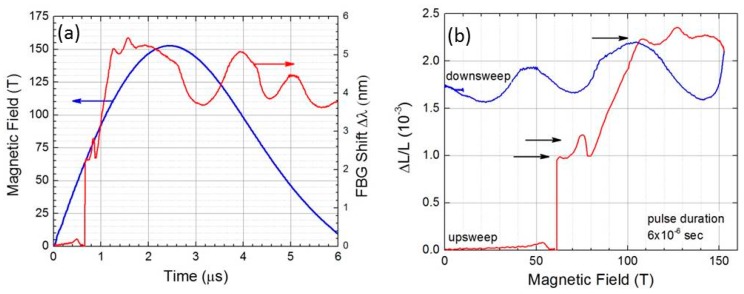
Axial magnetostriction obtained in a destructive 6 microsecond single-turn coil type magnet, figure reproduced from [17]. (**a**). Magnetic field (left y-axis, blue line) and FBG shift (right y-axis, red line) vs. time. (**b**). Computed ΔL/L vs. magnetic field for magnetic field upsweep (red) and down sweep (blue). Spin transitions at 60 T and 75 T, as well as a previously unknown transition at ~106 T, are indicated by arrows.

We studied the insulating perovskite cobaltite LaCoO_3_, whose octahedrally-coordinated Co^3+^ (3d^6^) ions are natural candidates to explore field-induced transitions of the electronic configuration [10,14,16,17]. A small gap ≈ 12 meV separates the *s* = 0 spin singlet ground state (6 electrons in the t_2g_ orbitals) from the lowest excited magnetic configuration. Although the Co^3+^ ions are in their *s* = 0 state at low temperatures, thermal activation to a *s* ≠ 0 magnetic state occurs above 30 K, thus giving rise to a paramagnetic response. Even though considerable work has been carried out on this thermally-induced spin crossover, the spin value of the first excited multiplet is still controversial. Since spin-state transitions involve changes in the occupation of electronic 3*d* orbitals, large changes in ionic radius and bonding occur, which, in turn, can lead to large overall length changes. Thus dilatometry and magnetostriction are important tools for investigating spin-state transitions. Figure 8 shows the magnetostriction of LaCoO_3_ that is measured in a single-turn coil destructive magnet to 150 T. Two anomalies observed at 60 T and 75 T reproduce results obtained in a 100 T, 15 ms, non-destructive magnet. A third anomaly observed at ~106 T has not yet been fully identified. The data taken during magnetic field down sweep does not reproduce the anomalies, signaling the detachment of the sample from the fiber. A likely reason for this is the field-induced mechanical shockwave propagation along the sample. 

## 4. Experimental Challenges, Artifacts, and Strategies Used to Address Them

### 4.1. Temperature Effects

The optical fibers from which FBG are constructed are made of SiO_2_. The relatively weak coefficient of thermal expansion of SiO_2_ is advantageous to create a low background for temperature-dependent strain measurements. The refractive index is, however, a strong nonlinear function of temperature causing a measurable and reproducible shift in the Bragg wavelength. This effect makes FBGs useful as chemically inert thermometers over a large temperature range. The low thermal conductivity of silica, however, prevents a very rapid sensor response. FBG-based thermometry has found some success in hostile environment monitoring. 

The strong temperature dependence is also an obstacle for their use in thermal dilatometry, and attention must be paid to the analysis of temperature dependent strain [3]. The Bragg wavelength *λ_B_* of an FBG changes with strain and temperature according to:

(1) $$\frac{\Delta \lambda_{B}}{\lambda_{B}}=\left(1-p\right)\varepsilon +\left(\frac{1}{n}\frac{dn}{dT}\right)\Delta T;$$

where the photoelastic coefficient *p* = 0.22 describes the sensitivity to strain in the 1550 nm band, and does not significantly depend on the temperature. The change of refractive index with temperature *α_δ_* = (1/*n*) *dn*/*dT* can be quite significant at high temperatures, although below 40 K it becomes nearly zero.

The contributions to the total strain arise from the mechanical strain on the sample (*ε_m_*) and by thermal contraction of the sample (*ε_s_*) such that *ε* = *ε_m_* + *ε_s_*. The second term can be written as *ε_s_* = *α_s_ΔT*, where *α_s_* is the coefficient of thermal expansion of the sample, which includes a contribution from the thermal expansion of the fiber. The mechanical strain on the sample, as measured by an FBG, is therefore:

(2) $$\varepsilon_{m}=\frac{1}{k}\frac{\Delta \lambda_{B}}{\lambda_{B}}-\left(\alpha_{s}+\frac{\alpha_{\delta }}{k}\right)\Delta T;$$

where *k*^−1^ = (*1*−*p*)^−1^ = 1.28. This expression is useful in the investigation of strain imposed on the sample by the sample holder during the process of cooling or heating, but it requires a detailed knowledge of *α_s_* and *α_δ_*. Alternatively, if the sample is free of applied mechanical strain (only attached to the fiber in the probe, *ε_m_* = 0) the coefficient of thermal expansion can be expressed as:

(3) $$\alpha_{s}=\frac{1}{k}\left(\frac{1}{\Delta T}\frac{\Delta \lambda_{B}}{\lambda_{B}}-\alpha_{\delta }\right);$$

If a second FBG is included on the same fiber, near the first one and also free of external strain, it can be used as reference. If the thermal expansion of the fiber is *α_f_*, then the resulting equation can be written:

(4) $$\alpha_{s}=\alpha_{f}+\frac{1.28}{\Delta T}\left(\frac{\Delta \lambda_{B}}{\lambda_{B}}-\frac{\Delta \lambda_{B}^{ref}}{\lambda_{B}^{ref}}\right);$$

The temperature dependence of the refractive index is in this way eliminated.

**Figure 9:**
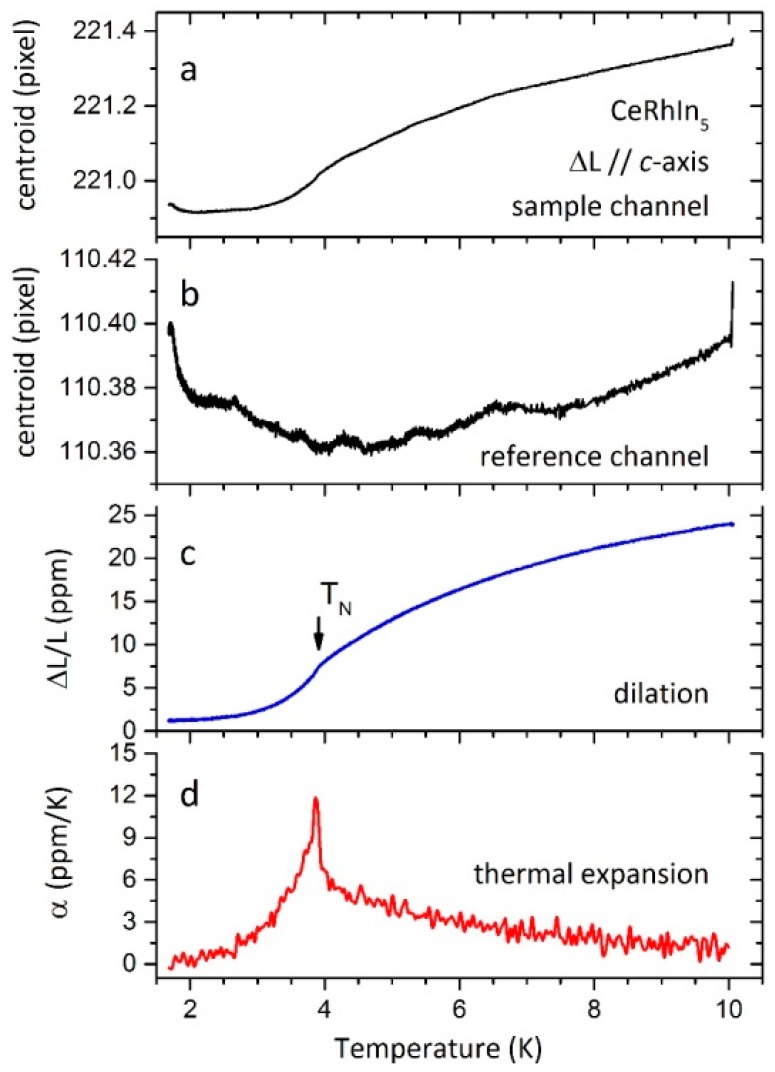
(**a**) FBG reflection centroid vs. temperature for the sample channel when a CeRhIn_5_ sample is glued to the fiber. (**b**) FBG reflection centroid vs. temperature for the reference channel. (**c**) Computed dilation *ΔL/L*(*T)* that is obtained by subtracting the reference channel from the sample channel and multiplying by a calibration constant. As it can be seen clearly here, the reference channel is essentially constant in the temperature range of the experiment, yet it is essential to remove systematic variations in the acquisition system that are common to both channels. (**d**) Computed coefficient of thermal expansion α(T). The noise level is <1 ppm. No correction was applied, neither by the thermal expansion of silica, or by strain transfer coefficient, with the notion of displaying just raw data. Note that the magnitude of the anomaly at the antiferromagnetic phase transition *T*_N_ ≈ 3.8 K is in good agreement with results using capacitive dilatometry [25].

There are essentially two alternative procedures at this point. When the material under study has a coefficient of thermal expansion *α_s_* >> *α_f_* in the temperature range of interest, then *α_f_* can be neglected. This approximation works very well at cryogenic temperatures when studying strongly correlated condensed matter systems. This is the favored approach of the authors of this work. Figure 9 shows an example of this approximation (i.e., neglecting *α_f_*) that is applied to the heavy-fermion material CeRhIn_5_. Even though the absolute values are small, the reference FBG picks up systematic variations that are common to the FBG attached to the sample.

On the other hand, if the material under study does not change much with temperature, then a calibration run must be carried out using a material of (small) known coefficient of thermal expansion in the temperature range of interest.

### 4.2. Magnetic Forces

Magnetically anisotropic samples experience a net torque in an applied magnetic field that is equal to the cross product of magnetization and applied magnetic field *τ* = ***M*** × ***H***. A finite torque is present when the sample magnetic moment ***M*** is not aligned with the applied magnetic field ***H***. A force can also occur in non-uniform applied magnetic fields. Torque or force on the sample can in turn cause an undesired extrinsic strain to be measured (a pervasive problem present in piezo-cantilever and capacitive dilatometers as well) or even the mechanical failure of the optical fiber. A characteristic feature of undesired torque or force effects contaminating magnetostriction data is a measured *ΔL/L ∝ H* when the sample magnetization is saturated, i.e., ***M****(H)* = constant. Here, the options to minimize torque-related artifacts include reducing the sample dimensions to needle-shaped samples, and improving applied magnetic field homogeneity as much as possible. 

### 4.3. Artifacts Due to Magnetic Field-Induced Faraday Rotation in the Optical Fiber

Faraday rotation (FR) refers to the polarization rotation of linearly-polarized light upon a passage through an optically active material. It arises when a material’s indices of refraction for right and left circularly polarized light are different. While only some materials are intrinsically gyrotropic (such as sugar water) and can induce Faraday rotation even at zero applied magnetic fields, essentially all materials exhibit some degree of optical activity (and therefore FR) in an applied magnetic field. The amount of FR that is induced (per unit field and per unit length of material) is characterized by a material’s wavelength-dependent Verdet constant. The Verdet constant for silica fibers at 1550 nm is approximately 0.8 rad/Tm. 

In principle, the center wavelength of the light that is reflected by a FBG is independent of the incident light polarization. In practice, however, any transverse strain gradient on the FBG (arising, for example, from the fact that the FBG is typically glued on only one side to a sample [5,45,46]) can lead to a slight difference in the reflected spectrum depending on whether the incident light is polarized along the transverse strain gradient, or orthogonal to it. Consider, then, the case where the incident light is partially linearly polarized (e.g., the output from most SLEDs is partially linearly polarized). During a high magnetic field pulse, the polarization direction of the light incident on the FBG will rotate (by many radians) due to Faraday rotation in the fiber. This will slightly modulate the center frequency of the reflected spectrum, giving an oscillatory artifact in the detected signal [45,46]. 

Oscillations due to FR can almost be entirely mitigated by using a polarization scrambler at the output of the light source before the light is sent to the FBG. Later iterations of the initial setup [6] utilize a polarization scrambler. In early experiments that did not use a polarization scrambler, however, these oscillatory artifacts posed a significant problem, because their influence could easily overwhelm smaller changes due to the true magnetostriction. We found, however, that in cases where a single monolithic magnet coil was used, the artifacts due to FR were sinusoidal with the applied magnetic field and had constant amplitude, and as such could be modeled and successfully subtracted from the data [12]. In contrast, when multi-stage magnets (such as our 100 T multi-shot magnet) were employed this procedure was less successful, because the different stages of the magnet, which are energized at different times, exhibit very different fringe fields, and are therefore induced very different time- and field-dependent Faraday rotations. 

**Figure 10:**
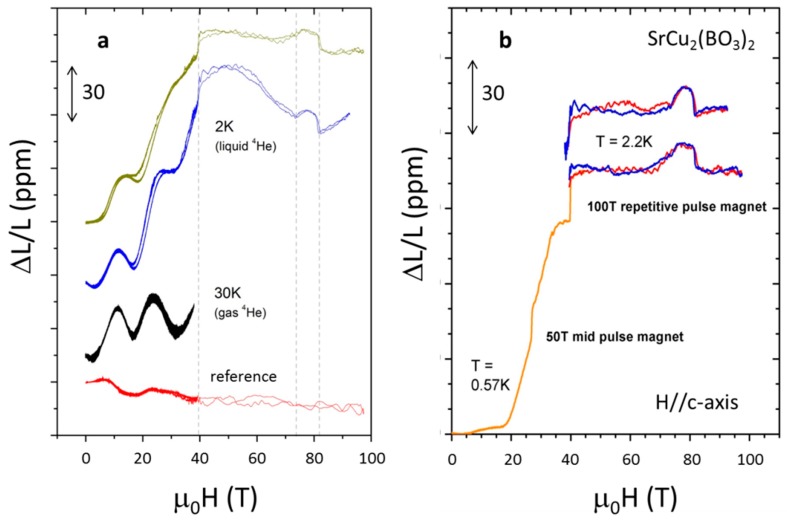
Magnetostriction *ΔL/L* vs. magnetic field of a SrCu_2_(BO_3_)_2_ single crystal measured in a 100 T magnet at cryogenic temperatures at the National High Magnetic Field Laboratory at Los Alamos National Laboratory [11]. (**a**) Magnetostriction measurement performed without a polarization scrambler in the experimental setup. The effects of Faraday rotation are evident in the oscillatory nature of data; (**b**) Magnetostriction data composed of results obtained in two different magnets, the 50 T mid pulse magnet for H < 45 T and the 100 T magnet for 35 < *H* < 97 T, after correcting for the effects of Faraday rotation. Red line is magnetic field up sweep, blue line is magnetic field down sweep.

The effect of FR can be clearly seen in Figure 10a, showing magnetostriction data taken in a non-destructive 100 T pulsed magnet. The sample under study, SrCu_2_(BO_3_)_2_ (Section 3.1) displays magnetization plateaus when a magnetic field large enough to close the spin gap (*H* > 20 T) is applied [11]. Figure 10b shows some of the same traces where (i) the low field traces were replaced by data taken in a single-coil 50 T mid pulse magnet where the FR effect could be simulated mathematically and subtracted, and (ii) the FR effect was subtracted from the high field (*H* > 50 T) data that was collected during the ramping of the single innermost coil in the 100 T magnet. The Faraday rotation artifact, as demonstrated here, can be quite detrimental to the sensitivity of the method and it is desirable to prevent it. 

### 4.4. Strain Measurements on Small Samples

An unavoidable constraint in the measurement of the magnetostriction of materials in high magnetic fields is the small size of samples under study. The availability of large high-quality single crystals; the detrimental effects of eddy current heating in metallic samples or magnetocaloric effects in magnetic insulators; and, magnetic torque effects put limits to the sample dimensions. Typical sample dimensions, hence, do not exceed a few millimeters in length and a fraction of a square millimeter in cross section. It is natural then, to question whether such small specimens can effectively drive the length of silica fibers ‘at will’, and whether any significant strain is imposed on materials by the fiber during cool-down to cryogenic temperatures due to differential thermal contraction. 

Here, we present a very simple yet practical analysis. The governing equations are based on Hook’s law, in which applied stress is linearly coupled to the strain. The coupling constants are the tensile modulus (also known as Young’s modulus) of the sample (*E_s_*) and the fiber (*E_f_*). When external parameters (*T* or *H*) change, and the change is small enough to assume a constant *E*, the sample under study dilates according to its coefficient of thermal expansion (*α*) or magnetostriction coefficient (λ). The force *F* necessary to bring the sample back to its original dimensions is given by F=(ΔL/L)×E×A, where *ΔL/L* is the relative deformation and *A* is the cross section. It is easy to show that the condition that must be fulfilled to justify neglecting the force imposed by the optical fiber on the sample during cooldown or application of a magnetic field is

(5) $$A_{s}\gg \left(E_{f}/E_{s}\right)\times A_{f},$$

where the subscripts refer to sample (*s*) and fiber (*f*). The elastic modulus for fused silica is approximately 70 GPa, roughly the value of most common metals, and the telecom-type 0.125 mm diameter fiber cross section is *A_f_* ≈ 0.012 mm^2^. Consequently, a bar-shaped metallic sample 0.5 mm on the side (cross section 0.25 mm^2^) fulfills Equation (5) quite satisfactorily. The elastic modulus of most solids, including fused silica, does not change significantly with temperature [<5%], so these considerations apply to the broad temperature range from 0.5 K to room temperature. Although the effect of magnetic fields on silica fibers is negligible, magnetic materials often become stiffer as a function of magnetic field and, as a consequence, even less susceptible to the force that is imposed by the fiber. Of course, there are soft organic materials that are more challenging to measure than simple metals. Equation (5), however, should suffice to estimate the required sample cross section to be used. 

Another rather useful quantitative analysis is to estimate the total applied force by the fused silica fiber on the sample due to differential thermal contraction from room temperature to cryogenic temperatures given by the formula

(6) $$F_{f}=E_{f}A_{f}\left(\alpha_{s}-\alpha_{f}\right)\Delta T,$$

where *α_s_* and *α_f_* are the coefficients of thermal expansion of the sample and fiber, respectively. In the case of copper for example, with α_Cu_ = 17 ppm/K, attached to a silica fiber (α_SiO2_ = 0.5–1 ppm/K) the force applied by the fiber on the sample is estimated to be smaller than 3 N for a temperature span of 200 K. As a comparison, the spring in a classical capacitive dilatometer for cryogenic temperatures, as the one described by Kuechler et al. [32,33] exerts a force estimated in 5 N on the samples under study. The main difference between these two methods is that while the force is compressive in the case of a capacitive dilatometer, it is tensile in the case of FBG grating dilatometry (except for materials that expand on cooling, in which case it is also compressive). Further, because the force is applied to the sample side, it can be non-uniform and cause birefringence-related artifacts [5,45,46]. 

Perhaps one of the most important issues that arises when an FBG-furbished optical fiber is attached to the surface of a material is ‘*shear lag*’, i.e., the interface mechanical limitations to fully transfer the strain. A number of analytical and experimental studies, by various authors, focus on this problem [47,48,49,50,51,52,53]. A model proposed to estimate the effects of shear lag in the limit where the elastic moduli of fiber coating and adhesive (acrylate glue) are negligible (only the shear moduli for these are considered) estimates the strain transfer coefficient *K_st_* as a function of the position along the fiber:

(7) $$K_{st}=\frac{\varepsilon_{f}\left(x\right)}{\varepsilon_{s}}=\frac{1-cosh\left(k_{2}x\right)/cosh\left(k_{2}l/2\right)}{k_{1}};$$

where

(8) $$k_{1}=1+\frac{\pi r_{f}^{2}}{2hr_{p}}\frac{E_{f}}{E_{s}};$$

and

(9) $$k_{2}=\sqrt{k_{1}\cdot \frac{2r_{p}}{\pi r_{f}^{2}E_{f}}\cdot \int_{0}^{\frac{\pi }{2}}{\left[\frac{r_{p}\left(1-\sin\theta \right)}{G_{a}}+\frac{r_{p}}{G_{p}}ln\left(\frac{r_{p}}{r_{f}}\right)\right]}^{-1}d\theta }$$

**Figure 11:**
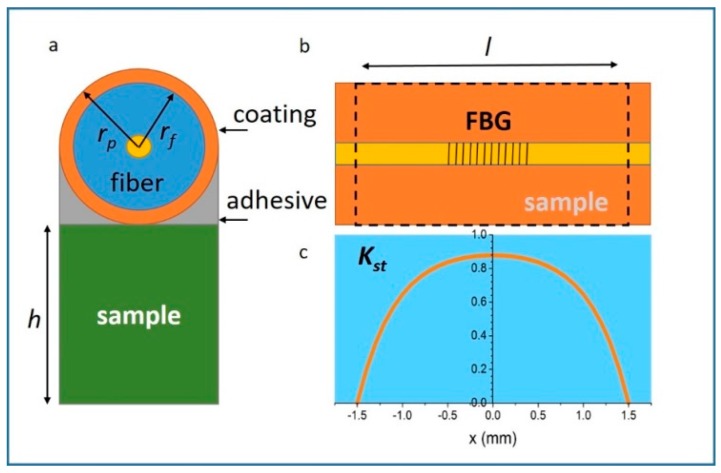
Schematics of a model to estimate the strain transfer (**a**) Cross-section of fiber plus sample ensemble for strain measurement. Here *h* is the sample thickness, *r_p_* is the coating radius, *r_f_* is the fiber cladding radius. (**b**) Fiber top view, indicating the sample length *l*, with respect to FBG position. (**c**) Strain transfer coefficient *K_st_* plotted as a function of position *x* on the fiber, computed using Equation (3) with parameters in Table 1.

**Figure 12:**
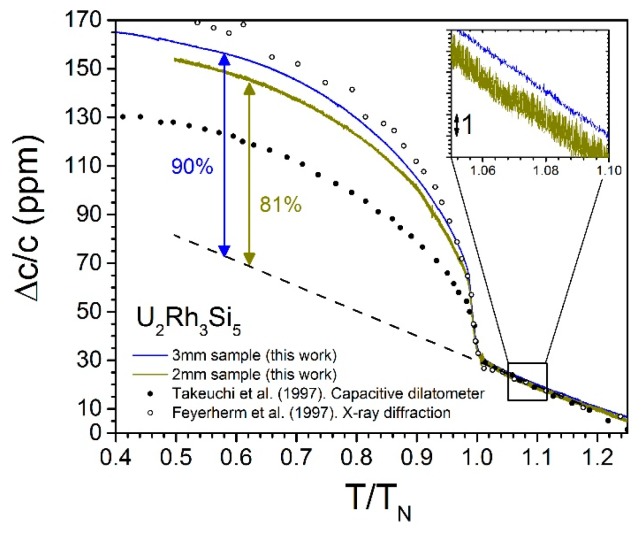
*c*-axis dilation Δ*c/c* vs. reduced temperature *T/T*_N_, where *T*_N_ = 25.5 K, for two samples of U_2_Rh_3_Si_5_ of identical cross section and different lengths, 2 mm (green) and 3 mm (blue), respectively, measured with a 0.5 mm long FBG. The magnitude of the effect of entering the antiferromagnetic phase for *T* < *T*_N_ is smaller for the smaller sample, with larger scattering (see inset). Data taken with a capacitive dilatometer (•, Takeuchi et al. [54]) and inferred from x-ray diffraction (○, Feyerherm et al. [55]) are included for comparison. The difference in magnitude between FBG and X-ray data is satisfactorily explained by the strain transfer coefficient given by Equation (3) when using the parameters listed in Table 1. Inset. Effect of the sample length on the measurement sensitivity. The scattering is enhanced in the short (2 mm long) sample, likely due to inhomogeneous strain transfer that translates to a broader reflection peak, as described by Equation (3) and displayed in Figure 13.

Using values in Table 1 and Equations (7)–(9), we obtain the strain profile along the fiber, as displayed in Figure 11. The x-axis origin is in the center of the FBG. The symmetric curve illustrates how the strain transfer drops monotonically towards both ends of the sample. Ideally, the FBG should be short enough (or the sample long enough) to occupy the central region of the sample, where the transfer can be considered relatively uniform. The drawback is that shorter FBGs lead to a reduction in sensitivity. We discuss this in more detail later. Figure 12 shows an example of two samples of different length measured using the same length of FBG. The small differences in the results can be modelled well using Equation (7).

The consequence of using a long FBG that extends beyond the uniform center is the detection of an asymmetric reflection peak. Equivalently, the observation of an asymmetric peak after cool down or during a magnetic field excursion is the first hint of a less than optimal choice of FBG length, fiber coating, sample dimensions, and adhesive. The data obtained in this way can, nevertheless, still be useful to detect temperature- or field-induced phase transitions that do not require a quantitative analysis of the magnetostriction coefficient.

### 4.5. Impact of FBG Length on ΔL/L Sensitivity

**Figure 13:**
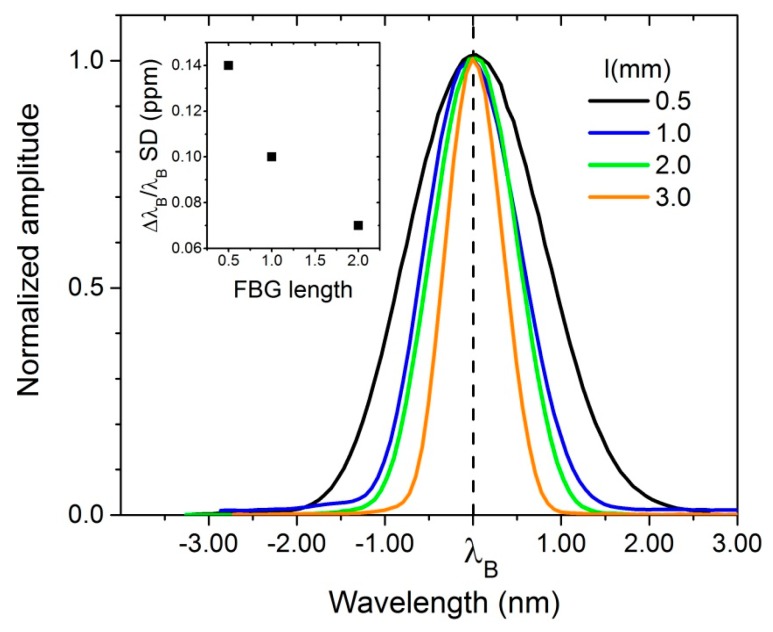
FBG reflections at *λ_B_* from sensors of different lengths (0.5, 1.0, 2.0 and 3.0 mm) manufactured by Technica SA, relative to *λ_B_* = 1550 nm. Inset. The standard deviation (SD) for the peak position obtained using a center-of-mass algorithm was obtained over a data acquisition period of one minute, and from it, *Δλ_B_/λ_B_* is computed. As expected, narrower reflections lead to an increased sensitivity when other parameters, such as intensity etc., are kept constant. Narrower peaks, however, require longer samples in the experiment.

A longer FBG produces a narrower reflected spectrum. Consequently, its position can be determined with higher precision resulting in smaller experimental error. This effect is demonstrated in Figure 13, where the reflection spectra for FBGs of different lengths ranging from 0.5 mm to 3 mm are displayed. Measurements performed at room temperature at a data acquisition rate of several kHz for a period of one minute were used to compute the standard deviation and the uncertainty in the position *Δλ_B_/λ_B_*. The uncertainty is found to double for an FBG four times shorter. 

**Figure 14:**
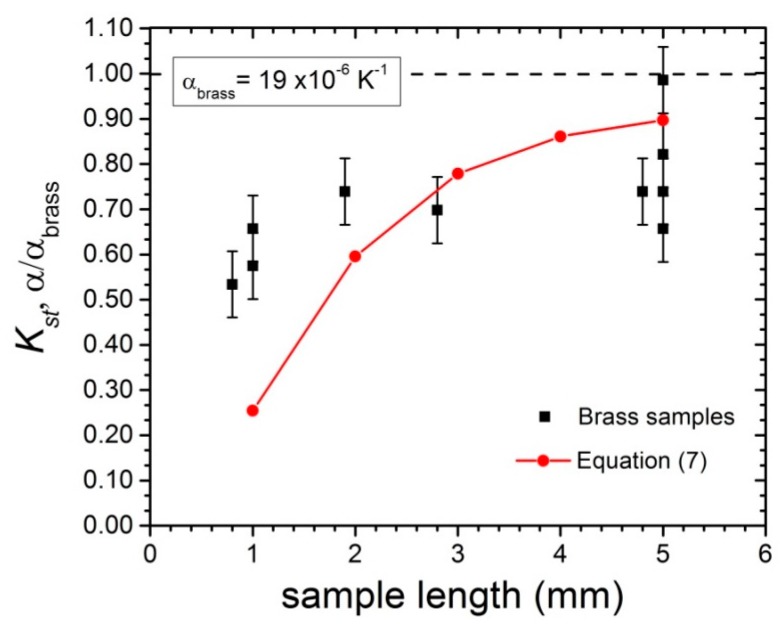
Strain transfer coefficient computed using Equation (7) (red circles), and measured coefficient of thermal expansion divided by α_brass_ = 19 ppm/K (black squares). The analytic solution fails to reproduce the experimental data in the 1 mm-long sample limit.

In order to test Equation (7), we carried out a series of simple experiments to estimate the strain transfer coefficient on brass samples with approximately 1 mm^2^ cross section and different lengths close to room temperature. The samples were glued to the FBG (0.5 mm long) with Pattex^®^ gel-type acrylate, and we measured the dilation between two fixed temperature points (water-ice coexistence and boiling water). We used our 46 kHz line scan array, as described in Section 2.2. The results are displayed in Figure 14. We find that Equation (7), using the room temperature recommended values *G_p_* = 2 GPa and *G_s_* = 25 MPa, clearly underestimates the strain transfer *K_st_* for small samples (<1 mm), while the results for 2–5 mm long samples show somewhat closer agreement (increased scattering makes it difficult to be more conclusive). Note that much better agreement is found at cryogenic temperatures (as shown in Figure 9 and Figure 12), possibly due to improved fiber coating and adhesive performances. Further investigations over a broader temperature range, using different adhesives, are necessary to reach definitive conclusions.

## 5. Conclusions

We present a review of three different experimental approaches to single mode SiO_2_ FBG dilatometry studies of materials in extreme conditions of high magnetic fields and low temperatures. A commercially off-the-shelf apparatus, the Hyperion^®^ model si155, is used to interrogate FBGs when magnetic fields are produced in a continuous fashion, achieving a strain resolution approaching *ΔL/L* ≈ 10^−8^ in the best cases. A 46 kHz setup based on an InGaAs line array camera is used in cases where the sample is exposed to millisecond long magnetic fields, achieving typical resolutions *ΔL/L* ≈ 10^−7^–10^−5^, depending on the type of magnet used. Finally, a time-delay dispersive pulsed laser approach has been implemented for the most demanding sample environments in microsecond-long magnetic field pulses that are produced by a single-turn type magnet. The strain sensitivity achieved in this case is better than 10^−4^. We also discuss typical shortcomings and artifacts when measuring mm-long samples in pulsed magnetic fields at cryogenic temperatures, and complete a quantitative evaluation of analytic models that re available in the literature. We conclude that small samples in the mm range perform better than predicted by available models. Additional studies performed with a broader selection of adhesives, and sample lengths, is under way.

## Figures and Tables

**Table 1:** Physical quantities and dimensions used for the computation of the strain transfer coefficient *K_st_* in Figure 11.

Description	Symbol	Value
Sample elastic modulus	*E_s_*	100 GPa
Fiber elastic modulus	*E_f_*	70 GPa
Polyimide coating shear modulus	*G_p_*	8 Gpa *
Acrylate adhesive shear modulus	*G_a_*	50 Mpa *
Sample length	*l*	3 mm
Sample thickness	*h*	0.5 mm
Fiber radius	*r_f_*	62.5 μm
Coating radius	*r_p_*	75 μm
Coefficient of strain transfer at FBG center	*K_st_*	0.902

* The shear moduli used for coating and glue are those expected for cryogenic temperatures.
